# An Overview of Systematic Reviews of Moxibustion for Knee Osteoarthritis

**DOI:** 10.3389/fphys.2022.822953

**Published:** 2022-02-03

**Authors:** Shao Yin, Fengya Zhu, Zhao Li, Deya Che, Liuying Li, Jie Feng, Lu Zhang, Zhenyi Huo

**Affiliations:** ^1^Hospital of Chengdu University of Traditional Chinese Medicine, Chengdu, China; ^2^Traditional Chinese Medicine Department, Zigong First People's Hospital, Zigong, China

**Keywords:** moxibustion, knee osteoarthritis, overview, systematic reviews, methodological quality

## Abstract

**Background:**

Currently, many systematic reviews (SRs) of moxibustion as a treatment of KOA have been published. However, the evidence of different SRs of moxibustion to treat KOA has not been comprehensively evaluated.

**Aim:**

This overview aimed to evaluate the existing results and provide scientific evidence of the effectiveness and safety of moxibustion in the treatment of KOA.

**Methods:**

We conducted a comprehensive search of Embase, PubMed, Web of Science, Cochrane Library, SinoMed, CNKI, Wanfang, VIP, and other databases until October 31, 2021. A Measurement Tool to Assess Systematic Reviews 2 (AMSTAR-2) was used to assess the methodological quality of SRs. Preferred Reporting Item for Systematic Reviews and Meta-Analyses was used to evaluate the reporting quality, and the risk of bias in SRs was evaluated by ROBIS Tool. We used the Grading of Recommendations Assessment, Development and Evaluation (GRADE) tool to determine the strength of evidence and conducted a meta-analysis of the total effectiveness rate.

**Results:**

Finally, 10 qualified SRs were included, including 57 randomized controlled trials and 5,149 participants. All SRs evaluated by AMASTAR-2 had more than one critical deficiency, so all SRs were rated as critically low. In the PRISMA checklist, the manuscript structures of the included SRs were relatively complete. Including four SRs with a low risk of bias and six with a high risk of bias using the ROBIS tool. In GRADE, two items (6.25%) were rated as high quality, three (9.37%) as medium quality, 17 (53.12%) as low quality and 10 (31.25%) as very low quality. A re-meta-analysis showed that moxibustion and moxibustion combined treatment improved the total effectiveness rate in knee osteoarthritis (risk ratio = 1.17, 95% confidence interval 1.13–1.21, *P* < 0.001 and risk ratio = 1.13, 95% CI: 1.04–1.23, *P* < 0.001), with low heterogeneity (*I*^2^ = 36.3%, *P* = 0.020, and *I*^2^ = 0.0%, *P* = 0.956). A total of eight SRs reported adverse events, and no serious adverse events occurred in the moxibustion group and control group.

**Conclusion:**

Moxibustion seems to be effective in treating KOA. Four SRs reported 10 common discomfort symptoms caused by moxibustion, and these adverse events can spontaneously subside, even can be avoided, therefore, moxibustion for KOA appears to be safe. However, the reliability of the results is reduced by the high risk of bias of the original studies and the low methodological quality of SRs. Therefore, future studies should pay more attention to the quality of the original study and the evidence quality of the SRs to provide more powerful and scientific evidence of the effectiveness and safety of moxibustion treatment of KOA.

## Introduction

Osteoarthritis is the most common joint disease in the world, and knee osteoarthritis (KOA) is the most common type with a high disability rate (Vos et al., 2012), and its pathological features are mainly persistent knee pain and dysfunction and degenerative changes of the articular cartilage (Hunter and Bierma-Zeinstra, 2019). Globally, KOA is ranked as the 11th leading cause of disability, with a prevalence rate of 3.8%, and is higher in women than in men (Cross et al., 2014). The prevalence of symptomatic KOA in older Chinese people (≥60 years old) was 19.4% (Xiang and Dai, 2009). Known risk factors for KOA are aging, overweight or obesity, occupational exposure, joint damage, and genetic factors (Wallace et al., 2017; Snoeker et al., 2020). New evidence proves that low-density inflammation is a key mediator in the pathogenesis of OA (Robinson et al., 2016). However, the deeper causes of the high prevalence of KOA remain unclear.

At present, KOA is still incurable, and its primary treatment goals are to relieve pain, improve mobility and walking, improve the quality of life, and slow down its progress when possible (Michael et al., 2010). According to the recommendations of the European League against Rheumatism (EULAR), KOA should be treated conservatively, that is, with a combination of drugs and non-drugs and surgical treatment if necessary, and treatment should be individualized (Pendleton et al., 2000). Non-drug therapy is the basis of drug therapy and surgical treatment. Moxibustion, as a physical therapy, is widely used to treat KOA in Asian countries (Huang et al., 2012b), which is also recommended by the Chinese Medical Association Bone Science Branch (2007).

Evidence from two systematic reviews (SRs) (Li et al., 2016; Song et al., 2016) showed that patients with KOA who received moxibustion obtained greater benefit in pain relief and improved function than those who received conventional care or sham moxibustion. However, there is a gap in the evidence and methodological quality among SRs. Although SRs are important for guiding evidence-based clinical practice, low reporting quality and high-risk SR may mislead clinical decision making. An overview of SRs is a new research method used to assess the quality of multiple SRs and try to resolve inconsistencies in evidence (Smith et al., 2011; Pollock et al., 2016). At present, the scientific quality of different SRs on the moxibustion treatment of KOA has not been comprehensively evaluated. Therefore, we evaluated the existing results through an overview of these SRs and provided scientific evidence of the effectiveness and safety of moxibustion in the treatment of KOA.

## Methods

### Search Strategy

We conducted a comprehensive search of the following eight databases: Embase, PubMed, Web of Science, Cochrane Library, SinoMed databases, China National Knowledge Infrastructure, Wanfang and VIP, and selected eligible SRs that had been published as of October 31, 2021. The search terms mainly include “osteoarthritis, knee,” “osteoarthritis,” “knee osteoarthritis,” “KOA,” “OA,” “moxibustion,” “systematic review,” “systematic evaluation,” and “meta-analysis.” The search format was adjusted to suit different databases (Supplementary Material 1). In addition, we manually searched for relevant references for review articles.

### Inclusion Criteria

#### Study Design and Participants

SRs based on random control trails (RCTs), moreover, meta-analysis has been used as a statistical method in the SRs to analyze and summarize the results of the included studies. All patients were diagnosed with KOA, regardless of age, sex, course, or severity.

#### Study Intervention

Moxibustion is the main intervention, including all non-intrusive moxibustion (such as traditional moxibustion, indirect moxibustion, heat-sensitive moxibustion, and thunder-fire moxibustion), or moxibustion combined with other treatments.

#### Study Comparison

The studies have compared routine treatment, placebo (sham moxibustion or blank control) or therapies other than moxibustion.

#### Study Outcomes

The study included at least one of the following: total effectiveness rate, pain score, Western Ontario and McMaster Universities Osteoarthritis Index (WOMAC) scale, WOMAC pain score, Lysholm score, 36-Item Short Form Survey (SF-36) scale, and Lequesne index. Because these are the most widely used outcomes to observe the efficacy of KOA. The total effectiveness rate was a compound outcome and total effectiveness rate = (basically cured patients + markedly improved patients + improved patients)/total number of patients (Zhang, 1999).

### Exclusion Criteria

1. The main intervention was not moxibustion, or the intervention was invasive moxibustion, such as warm-needle moxibustion.
2. SRs of the comparison of different types of moxibustion.
3. Other types of research, such as animal experiments, protocols, conference papers, case reports, and guidelines.
4. Literatures with duplication of data and inaccessibility of the full text.

### Study Selection and Data Extraction

Two reviewers (SY and ZYH) conducted literature screening independently. All search results were imported into Endnote (X9.3) to remove duplicates, and inconsistent articles were then removed based on their titles and abstracts. Finally, the full text was read, and eligible SRs were included. Unresolved differences were resolved by a third reviewer (FYZ).

Two reviewers (JF and LZ) independently extracted the basic characteristics of the literature, including authors, year of publication, diagnostic criteria, sample size, intervention, comparison, outcomes, adverse effects, and methodological evaluation tool. Two reviewers cross-checked the extracted content and consulted a third reviewer (FYZ) for any differences.

### Assessment of SRs

Two reviewers (SY and ZL) used the following four evaluation tools: A Measurement Tool to Assess Systematic Reviews 2 (AMSTAR-2) (Shea et al., 2017; Tao et al., 2018), Preferred Reporting Item for Systematic Reviews and Meta-Analyses (PRISMA) (Liberati et al., 2009), ROBIS tool (Whiting et al., 2016), and Grading of Recommendations Assessment, Development, and Evaluation (GRADE) (Atkins et al., 2004). They independently evaluated the included SRs and then cross-checked. Any differences were resolved through negotiation and unresolvable consultation with a third reviewer (FYZ).

AMSTAR-2 is used to evaluate the methodological quality of the included SR. It contains 16 items, of which 2, 4, 7, 9, 11, 13, and 15 are key items. Then, an overall assessment of SR (high, medium, low, and critically low) is performed based on the evaluation of key items and non-critical items.

PRISMA is used to assess the quality of report, covering 27 items. Each item can be assessed as “yes,” “partial yes,” and “no,” and the rate is listed according to the evaluation of each item.

ROBIS is a tool used to evaluate the risk of bias (RoB) in SR. It is divided into three phases. The first phase is optional, and the second phase consists of four key areas: “study eligibility criteria,” “identification and selection of studies,” “data collection and study appraisal,” and “synthesis and findings.” The third phase is based on the evaluation of the four areas in the second stage for comprehensive evaluation, and the SRs are evaluated as “low risk,” “high risk,” and “unclear risk.”

GRADE is used to evaluate the quality of evidence for results based on five key factors: RoB, inconsistency, indirectness, imprecision, and publication Bias. The quality of evidence is rated as “high,” “moderate,” “low,” and “very low.”

### Strategy for Data Synthesis

In addition to the descriptive analysis of existing data, we re-analyzed the main outcome to observe the efficacy of moxibustion and combination therapy in the treatment of KOA. Stata15.1 was used in the data analysis, and dichotomous variables are represented by the risk ratio (RR) and 95 confidence interval (CI), if *P* < 0.05, it means there is a statistical significance. When there is obvious heterogeneity (*I*^2^ > 50%), the random-effects model should be used and to explore the source of heterogeneity. Funnel plot and Egger's test were used to detect publication bias, and sensitivity analysis was used to test the stability of the results.

## Results

### Results on Literature Search and Selection

**Figure 1:**
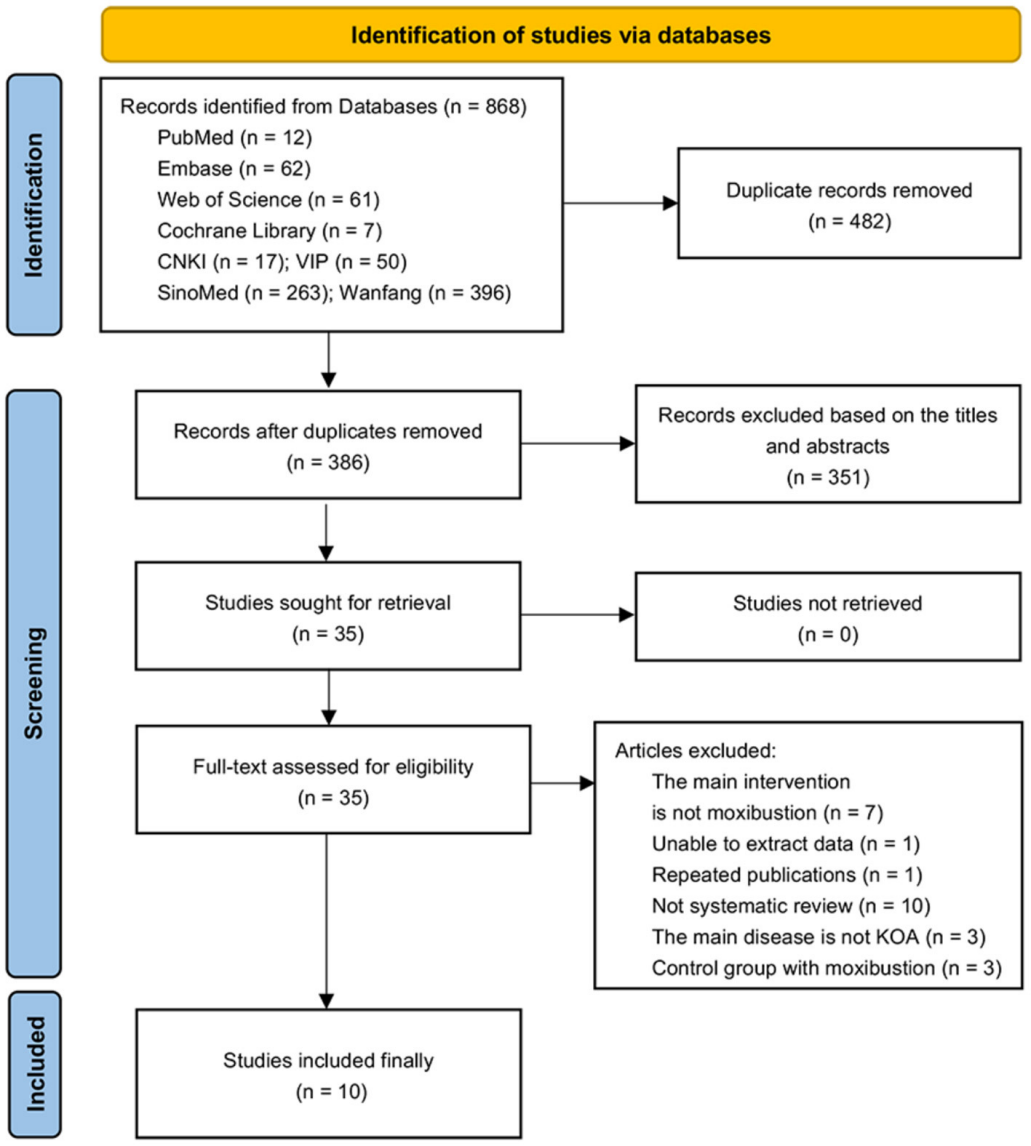
Flow chart of study selection.

A total of 868 records were retrieved from the database. After removing 482 duplicative items, 351 articles were screened according to the title and abstract, and the full texts of the 35 articles were then evaluated. Finally, 10 SRs of moxibustion treatment of KOA were included (Figure 1). The excluded articles and reasons for exclusions in “full-text assessed for eligibility” are shown in the (Supplementary Material 1).

### Characteristics of Included SRs

The 10 included SRs were published between 2016 and 2021, of which two were published in English and the remaining eight were published in Chinese. A total of 57 RCTs and 5,149 participants were included in this overview after duplicates removed (Supplementary Material 2). Each SR included 4–16 RCTs, with sample sizes of 634–1,593 participants. The diagnostic criteria vary: six SRs (Li et al., 2016, 2019; Song et al., 2016; Ma et al., 2017; Wang et al., 2017; Fan et al., 2018) used the diagnostic criteria of the American College of Rheumatology, one SR (Zhang et al., 2017) adopted the guiding principle of clinical research on new drugs in the treatment of KOA and the remaining three SRs (Lu et al., 2019; Deng et al., 2020; Zhang et al.) did not report the basis of diagnostic criteria. Four SRs (Li et al., 2016; Song et al., 2016; Zhang et al., 2017; Lu et al., 2019) reported adverse events in the treatment and control groups, three SRs (Li et al., 2019; Deng et al., 2020; Zhang et al.) only reported adverse events in the control group, one SR (Wang et al., 2017) showed no adverse events and two SRs (Ma et al., 2017; Fan et al., 2018) did not mention adverse events. Regarding the quality assessment of the original studies, the Cochrane risk of bias tool was used for seven SRs (Li et al., 2016, 2019; Song et al., 2016; Ma et al., 2017; Fan et al., 2018; Lu et al., 2019; Zhang et al.), the Jadad scale was used for two SRs (Wang et al., 2017; Zhang et al., 2017) and the Cochrane risk of bias tool plus Jadad scale was used for one SR (Deng et al., 2020). Detailed basic characteristics of the included SRs are shown in Table 1.

**Table 1:** Characteristics of the included SRs.

**Included studies**	**Language**	**Number of RCTs (participants)**	**Diagnostic criteria**	**Intervention (D/F)**	**Comparison (D/F)**	**Adverse effects (number of RCTs, E/C)**	**Methodological evaluation tool**	**Primary outcomes**	**Main conclusion**
Song et al. (2016)	English	13 (1,309)	ACR/G	Moxibustion 20–60 min (4–6 w/three times a week)	Inject sodium hyaluronate 2 ml (3–4 w/once a week)	Burn wounds, pruritus, fatigue, blisters, and skin flushing skin flushing, blisters. (3/1)	Cochrane risk of bias tool	①②	Moxibustion was not statistically different from oral drug in alleviating pain, improving function, and increasing response rate
					Diclofenac sodium 75 mg, celecoxib 200 mg, sham moxibustion (3–6 w/qd)				
Li et al. (2016)	English	4 (746)	ACR/G	Moxibustion 20–45 min (4–6 w/three times a week)	Sham moxibustion (4–6 w/three times a week)	Blisters, burn wounds, and skin flushing in local lesions, abnormal reactions (4/1)	Cochrane risk of bias tool	③④⑥	The administration of moxibustion can to some extent alleviate the symptoms of KOA
Wang et al. (2017)	Chinese	12 (843)	ACR/C/D/G	Moxibustion (Not reported)	Physical therapy, inject sodium hyaluronate, sham moxibustion (Not reported)	No adverse events	Jadad	①②⑤	Moxibustion is a safe and effective TCM therapy for knee osteoarthritis
Ma et al. (2017)	Chinese	7 (797)	ACR/G	Moxibustion 20 min (4–6 w/three times a week)	Celecoxib 200 mg (6 w/qd)	Not reported	Cochrane risk of bias tool	②③⑥	Moxibustion intervention can relieve the pain and improve the quality of life of patients with knee osteoarthritis
Zhang et al. (2017)	Chinese	10 (866)	G	Moxibustion (Not reported)	Diclofenac sodium (Not reported)	Stomach upset, nausea, stomach pain, rash, itching (1/1)	Jadad	①	The clinical effect of indirect moxibustion on KOA is better than that of non-steroidal anti-inflammatory painkillers
Fan et al. (2018)	Chinese	11 (935)	ACR/C/D/G	Moxibustion 30 min (4–6 w/qd)	Diclofenac sodium 75 mg, celecoxib 200 mg (3–6 w/qd)	Not reported	Cochrane risk of bias tool	①②③	Moxibustion is superior to drug therapy in knee osteoarthritis
Lu et al. (2019)	Chinese	7 (634)	Not reported	Moxibustion 30 min (4–6 w/qd)	Physical therapy, sham moxibustion, acupuncture, diclofenac sodium 75 mg (2–6 w/qd)	Blisters, burns, itching and fatigue, nausea, epigastric discomfort (2/2)	Cochrane risk of bias tool	①④⑤	Moxibustion improved WOMAC scale and Lysholm knee function score in KOA patients better than the control group, and the total effectiveness rate was significantly higher than the control group
Li et al. (2019)	Chinese	15 (1,207)	ACR/C/D/G	Moxibustion (Not reported)	Drug therapy (Not reported)	Nausea, stomachache, epigastric pain (0/2)	Cochrane risk of bias tool	①②③④⑤⑦	Moxibustion is a safe, effective and simple treatment for KOA
Deng et al. (2020)	Chinese	10 (987)	Not reported	Moxibustion (Not reported)	Diclofenac sodium, celecoxib, fenbid, traditional Chinese medicine (Not reported)	Emesis (0/1)	Cochrane risk of bias tool, Jadad	①②③⑦	Thunder-fire moxibustion at this stage of KOA clinical study may be a relatively safe treatment method
Zhang et al. (2021)	Chinese	16 (1,593)	Not reported	Moxibustion+ Comparison (Not reported)	Diclofenac sodium, celecoxib, fenbid, traditional Chinese medicine, arthroscopic treatment, electric acupuncture (2–6 w/Not reported)	Nausea, stomachache (0/1)	Cochrane risk of bias tool	①②③④	Thunder-fire moxibustion is better than other treatments

*①, Total effectiveness rate; ②, Pain Score; ③, WOMAC Scale; ④, WOMAC Pain Score; ⑤, Lysholm Score; ⑥, SF-36 Scale; ⑦, Lequesne Index.*

*D, Duration; F, Frequencies; w, week; qd, once a day; ACR, American College of Rheumatology; C, Criteria for the Diagnosis and Therapeutic Effect of TCM Diseases; D, Diagnostic Criteria of the Osteoarthritis Treatment Guide; G, guiding principle of clinical research on new drugs in the treatment of knee osteoarthritis score*.

### Methodological Assessment

AMASTAR-2 was used to assess the methodological quality of the SRs included in this study, and all SRs were rated as having critically low quality due to more than one serious deficiency in critical items 2, 4, 7, 9, 11, 13, and 15 and multiple deficiencies in non-critical items. In critical items, nine SRs (Li et al., 2016, 2019; Song et al., 2016; Wang et al., 2017; Zhang et al., 2017; Fan et al., 2018; Lu et al., 2019; Deng et al., 2020; Zhang et al.) did not specify the registration in advance research project (item 2), all SRs did not provide a comprehensive literature search strategy (item 4) and exclusion and exclusion criteria (item 7). Five SRs (50%) (Song et al., 2016; Ma et al., 2017; Fan et al., 2018; Li et al., 2019; Deng et al., 2020) selected appropriate effect sizes and statistical methods during the meta-analysis and investigated the sources of heterogeneity and reasonable explanations (item 11). Six SRs (60%) (Ma et al., 2017; Zhang et al., 2017; Fan et al., 2018; Li et al., 2019; Deng et al., 2020; Zhang et al.) reported publication bias (item 15). Of the non-critical items, only one SR (10%) (Zhang et al.) explained the reasons for the type of design included in the study (item 3), eight SRs (80%) (Li et al., 2016; Song et al., 2016; Ma et al., 2017; Zhang et al., 2017; Fan et al., 2018; Lu et al., 2019; Deng et al., 2020; Zhang et al.) mentioned that two people independently performed literature screening (item 5) and data extraction (item 6) and one SR (10%) (Lu et al., 2019) reported the source of funding for the included RCTs (item 10). None of the SRs assessed the potential effect of RoB in a single RCT (item 12), five SRs (50%) (Song et al., 2016; Ma et al., 2017; Fan et al., 2018; Li et al., 2019; Deng et al., 2020) had no heterogeneity or did not explain the heterogeneity reasonably (item 14) and two SRs (20%) (Li et al., 2016; Song et al., 2016) claimed no conflict of interest and identified funding sources (item 16). Detailed results are shown in Table 2.

**Table 2:** AMSTAR-2 for methodological quality of the included SRs.

**Included studies**	**AMASTAR 2**																**Quality**
	**Item 1**	**Item 2**	**Item 3**	**Item 4**	**Item 5**	**Item 6**	**Item 7**	**Item 8**	**Item 9**	**Item 10**	**Item 11**	**Item 12**	**Item 13**	**Item 14**	**Item 15**	**Item 16**	
Song et al. (2016)	Y	**N**	N	**PY**	Y	Y	**N**	PY	**Y**	N	**Y**	N	**Y**	Y	**N**	Y	Critically low
Li et al. (2016)	Y	**N**	N	**PY**	Y	Y	**N**	Y	**Y**	N	**N**	N	**Y**	N	**N**	Y	Critically low
Wang et al. (2017)	Y	**N**	N	**N**	N	N	**N**	PY	**PY**	N	**N**	N	**Y**	N	**N**	N	Critically low
Ma et al. (2017)	Y	**PY**	N	**PY**	Y	Y	**N**	PY	**Y**	N	**Y**	N	**Y**	Y	**Y**	N	Critically low
Zhang et al. (2017)	Y	**N**	N	**PY**	Y	Y	**N**	PY	**Y**	N	**N**	N	**Y**	N	**Y**	N	Critically low
Fan et al. (2018)	Y	**N**	N	**PY**	Y	Y	**N**	Y	**Y**	N	**Y**	N	**Y**	Y	**Y**	N	Critically low
Lu et al. (2019)	Y	**N**	N	**PY**	Y	Y	**N**	Y	**Y**	Y	**N**	N	**Y**	N	**N**	N	Critically low
Li et al. (2019)	Y	**N**	N	**PY**	N	N	**N**	PY	**Y**	N	**Y**	N	**Y**	Y	**Y**	N	Critically low
Deng et al. (2020)	Y	**N**	N	**N**	Y	Y	**N**	PY	**Y**	N	**Y**	N	**Y**	Y	**Y**	N	Critically low
Zhang et al. (2021)	Y	**N**	Y	**PY**	Y	Y	**N**	PY	**Y**	N	**N**	N	**Y**	N	**Y**	N	Critically low
YES [*n* (%)]	10 (100)	**0 (0)**	1 (10)	**0 (0)**	8 (80)	8 (80)	**0 (0)**	3 (30)	**9 (90)**	1 (10)	**5 (50)**	0 (0)	**10 (100)**	5 (50)	**6 (60)**	2 (20)	

*Y, yes; PY, partial yes; N, no. Bold values are critical items of AMSTAR-2*.

### Reporting Quality

The quality assessment results of the PRISMA checklist are shown in Table 3. The manuscript structures of the SRs included in this overview were relatively complete. The title, introduction and discussion sections have good integrity (100%); however, other sections have some deficiencies. For example, item 5 (protocol and registration), item 8 (search), item 15 (risk of bias across studies), and item 16 (additional analyses) in the abstract (structured summary) and methods and item 23 (additional analysis) in results, all reported incomplete or unreported issues (<50%).

**Table 3:** The quality assessment results of the PRISMA checklist.

**Section/ topic**	**Items**	**Included studies**										**YES [*n* (%)]**
		**Song et al. (2016)**	**Li et al. (2016)**	**Wang et al. (2017)**	**Ma et al. (2017)**	**Zhang et al. (2017)**	**Fan et al. (2018)**	**Lu et al. (2019)**	**Li et al. (2019)**	**Deng et al. (2020)^**)**^**	**Zhang et al. (2021)**	
Title	Item 1	Y	Y	Y	Y	Y	Y	Y	Y	Y	Y	10 (100)
Abstract	Item 2	PY	PY	PY	PY	PY	PY	PY	PY	PY	PY	0 (0)
Introduction	Item 3	Y	Y	Y	Y	Y	Y	Y	Y	Y	Y	10 (100)
	Item 4	Y	Y	Y	Y	PY	Y	Y	Y	Y	Y	9 (90)
Methods	Item 5	N	N	N	PY	N	N	N	N	N	N	0 (0)
	Item 6	Y	Y	Y	Y	Y	Y	Y	Y	Y	Y	10 (100)
	Item 7	Y	Y	PY	Y	PY	Y	Y	PY	PY	PY	5 (50)
	Item 8	PY	PY	PY	Y	PY	Y	PY	Y	PY	Y	4 (40)
	Item 9	Y	Y	Y	Y	Y	Y	Y	Y	Y	Y	10 (100)
	Item 10	Y	Y	N	Y	Y	Y	Y	PY	PY	PY	6 (60)
	Item 11	Y	Y	Y	Y	PY	Y	Y	Y	Y	Y	9 (90)
	Item 12	Y	Y	Y	Y	Y	Y	Y	Y	Y	Y	10 (100)
	Item 13	Y	Y	Y	Y	Y	Y	Y	Y	Y	Y	10 (100)
	Item 14	Y	Y	Y	Y	Y	Y	Y	Y	Y	Y	10 (100)
	Item 15	N	N	N	Y	N	N	N	N	Y	Y	3 (30)
	Item 16	Y	Y	N	N	N	N	N	N	Y	Y	4 (40)
Results	Item 17	Y	Y	PY	PY	Y	Y	PY	Y	Y	Y	7 (70)
	Item 18	Y	Y	Y	Y	PY	Y	Y	Y	Y	Y	9 (90)
	Item 19	Y	Y	Y	Y	Y	Y	Y	Y	Y	Y	10 (100)
	Item 20	Y	Y	Y	Y	Y	Y	Y	Y	Y	Y	10 (100)
	Item 21	Y	Y	Y	Y	Y	Y	Y	Y	Y	Y	10 (100)
	Item 22	N	N	N	Y	Y	Y	N	Y	Y	Y	6 (60)
	Item 23	Y	N	N	N	N	Y	N	N	Y	Y	3 (30)
Discussion	Item 24	Y	Y	Y	Y	Y	Y	Y	Y	Y	Y	10 (100)
	Item 25	Y	Y	Y	Y	Y	Y	Y	Y	Y	Y	10 (100)
	Item 26	Y	Y	Y	Y	Y	Y	Y	Y	Y	Y	10 (100)
Funding	Item 27	N	Y	N	N	Y	Y	Y	Y	Y	Y	7 (70)

*Y, yes; PY, partial yes; N, no*.

### RoB

**Figure 2:**
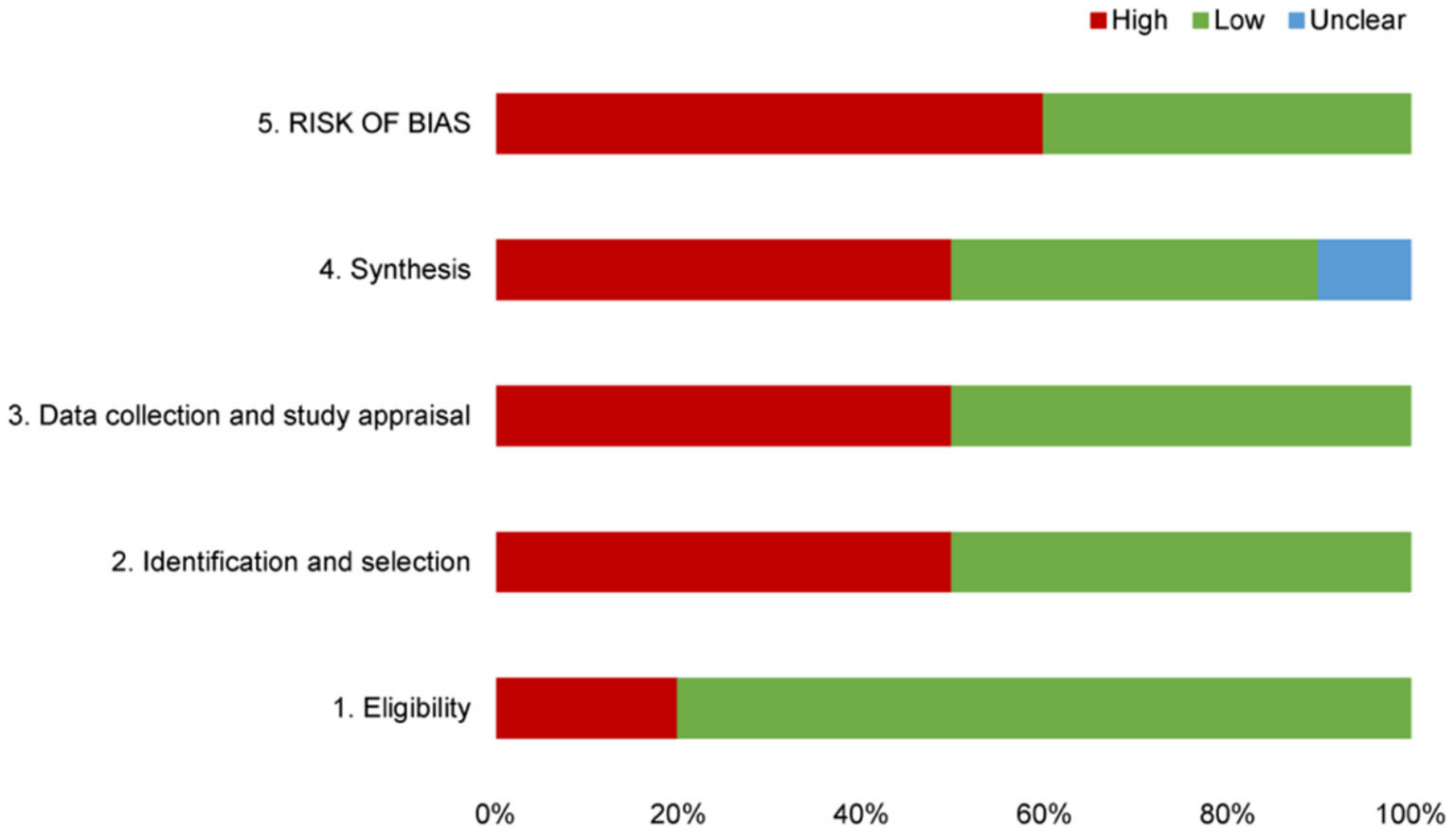
Graphical presentation of risk of bias of the included SRs.

The RoB assessments of SRs included in this overview are shown in Table 4 and Figure 2. Four SRs (40%) were rated as low risk, and six SRs (60%) were rated as high risk through the comprehensive assessment in phase 3. Failure to properly explain and deal with the RoB may lead to high RoB in SR.

**Table 4:** Tabular presentation for ROBIS results.

**Review**	**Phase 2**			**Phase 3**	
	**(1) Study eligibility criteria**	**(2) Identification and selection of studies**	**(3) Data collection and study appraisal**	**(4) Synthesis and findings**	**Risk of bias in the review**
Song et al. (2016)	☺	☺	☹	☺	☺
Li et al. (2016)	☺	☹	☺	?	☹
Wang et al. (2017)	☺	☹	☹	☺	☹
Ma et al. (2017)	☺	☺	☺	☹	☺
Zhang et al. (2017)	☺	☹	☺	☹	☹
Fan et al. (2018)	☺	☺	☺	☺	☺
Lu et al. (2019)	☹	☹	☹	☹	☹
Li et al. (2019)	☺	☺	☺	☹	☺
Deng et al. (2020)	☹	☺	☹	☺	☹
Zhang et al. (2021)	☺	☹	☹	☹	☹

*☺, low risk; ☹, high risk; ?, unclear risk*.

### Quality of Evidence

Ten SRs included a total of 32 outcomes. The results showed that two (6.25%) were rated as high quality, three (9.37%) as moderate quality, 17 (53.12%) as low quality and 10 (31.25%) as very low quality. RoB (29/32, 90.62%), inconsistency (10/32, 31.25%), and publication bias (26/32, 81.25%) were the main factors for demoting results. Related results are shown in Table 5.

**Table 5:** GRADE for quality of evidence profile.

**Included studies**	**Outcomes**	**No. of RCTs (participants, E/C)**	**Certainty assessment**					**Effect estimate (95% CI)**	***P-*value**	**Quality of evidence**
			**Risk of bias**	**Inconsistency**	**Indirectness**	**Imprecision**	**Publication bias**			
Song et al. (2016)	Total effectiveness rate	6 (272/268)	Serious^a^	Not serious	Not serious	Not serious	Serious^d^	RR 1.09 (1.03, 1.17)	0.005	Low
	Pain score	4 (179/160)	Serious^a^	Not serious	Not serious	Not serious	Serious^d^	SMD −0.17 (−0.39, 0.05)	0.12	Low
Li et al. (2016)	WOMAC scale	2 (157/165)	Serious^a^	Not serious	Not serious	Serious^c^	Serious^d^	MD 17.63 (−23.15, 58.41)	0.40	Very low
	WOMAC pain score	2 (157/165)	Serious^a^	Not serious	Not serious	Serious^c^	Serious^d^	MD 13.45 (−26.99, 53.89)	0.51	Very low
	SF-36 scale	2 (171/177)	Serious^a^	Not serious	Not serious	Not serious	Serious^d^	MD 0.98 (−0.97, 2.93)	0.32	Low
Wang et al. (2017)	Total effectiveness rate	8 (292/288)	Serious^a^	Not serious	Not serious	Not serious	Serious^d^	RR 1.18 (1.10, 1.27)	<0.0001	Low
	Pain score	3 (124/107)	Serious^a^	Not serious	Not serious	Not serious	Serious^d^	MD −1.35 (−1.67, −1.02)	<0.0001	Low
	Lysholm score	3 (85/80)	Serious^a^	Not serious	Not serious	Not serious	Serious^d^	MD 1.45 (0.82, 2.08)	<0.0001	Low
Ma et al. (2017)	Pain score	4 (179/160)	Serious^a^	Serious^b^	Not serious	Not serious	Not serious	MD 0.64 (0.02, 1.27)	0.04	Low
	WOMAC scale	2 (171/177)	Not serious	Not serious	Not serious	Not serious	Not serious	MD 1.86 (1.50, 2.22)	<0.0001	High
	SF-36 Scale	2 (157/165)	Not serious	Not serious	Not serious	Not serious	Not serious	MD 0.94 (−0.04, 1.91)	0.06	High
Zhang et al. (2017)	Total effectiveness rate	10 (433/429)	Serious^a^	Not serious	Not serious	Not serious	Serious^d^	OR 3.26 (2.12, 5.02)	<0.0001	Low
Fan et al. (2018)	Total effectiveness rate	10 (439/437)	Serious^a^	Not serious	Not serious	Not serious	Serious^d^	RR 0.47 (0.33, 0.67)	None	Low
	Pain score	7 (298/281)	Serious^a^	Serious^b^	Not serious	Not serious	Serious^d^	SMD 1.53 (−2.11, −0.95)	None	Very Low
	WOMAC scale	2 (134/134)	Serious^a^	Serious^b^	Not serious	Not serious	Serious^d^	SMD −0.85 (−1.11, −0.59)	None	Very low
Lu et al. (2019)	Total effectiveness rate	5 (184/179)	Serious^a^	Not serious	Not serious	Not serious	Not serious	OR 3.68 (1.72, 7.87)	<0.05	Moderate
	WOMAC pain score	2 (133/138)	Not serious	Not serious	Not serious	Not serious	Serious^d^	MD −2.22 (−3.21, −1.24)	<0.05	Moderate
	Lysholm score	2 (70/63)	Serious^a^	Not serious	Not serious	Not serious	Serious^d^	MD −7.79 (4.21, 11.37)	<0.05	Low
Li et al. (2019)	Total effectiveness rate	11 (505/507)	Serious^a^	Not serious	Not serious	Not serious	Not serious	RR 1.21 (1.14, 1.28)	<0.0001	Moderate
	Pain score	7 (301/266)	Serious^a^	Serious^b^	Not serious	Not serious	Not serious	MD −2.71 (−4.90, 0.52)	0.02	Low
	WOMAC scale	4 (207/205)	Serious^a^	Serious^b^	Not serious	Not serious	Serious^d^	MD −6.79 (−12.35, −1.23)	0.02	Very Low
	WOMAC pain score	3 (203/199)	Serious^a^	Serious^b^	Not serious	Not serious	Serious^d^	MD −1.34 (−2.50, −0.17)	0.02	Very Low
	Lysholm score	1 (40/40)	Serious^a^	Not serious	Not serious	Not serious	Serious^d^	MD 12.13 (6.87, 17.39)	<0.01	Low
	Lequesne index	1 (30/30)	Serious^a^	Not serious	Not serious	Not serious	Serious^d^	MD −4.22 (−5.74, −2.70)	<0.01	Low
Deng et al. (2020)	Total effectiveness rate	9 (478/449)	Serious^a^	Not serious	Not serious	Not serious	Serious^d^	OR 3.19 (2.07,4.90)	<0.0001	Low
	Pain score	5 (205/206)	Serious^a^	Not serious	Not serious	Not serious	Serious^d^	MD −1.66 (−2.16, −1.16)	<0.0001	Low
	WOMAC scale	2 (104/104)	Serious^a^	Serious^b^	Not serious	Not serious	Serious^d^	MD −1.95 (−4.52,0.62)	0.14	Very Low
	Lequesne index	1 (46/46)	Serious^a^	Not serious	Not serious	Not serious	Serious^d^	MD −5.29 (−5.97, −4.61)	<0.0001	Low
Zhang et al. (2021)	Total effectiveness rate	12 (659/617)	Serious^a^	Not serious	Not serious	Not serious	Serious^d^	RR 1.13 (1.08, 1.18)	<0.0001	Low
	Pain score	7 (273/273)	Serious^a^	Serious^b^	Not serious	Not serious	Serious^d^	SMD −1.41 (−2.07, −0.75)	<0.0001	Very low
	WOMAC scale	4 (187/186)	Serious^a^	Serious^b^	Not serious	Not serious	Serious^d^	SMD −1.23 (−2.39, −0.08)	0.04	Very low
	WOMAC pain score	4 (182/180)	Serious^a^	Serious^b^	Not serious	Not serious	Serious^d^	SMD −0.91 (−1.47, −0.34)	0.002	Very low

*E, experimental group; C, control group.*

a *The risk of bias is unclear in most of the studies.*

b *The confidence interval overlap less, the heterogeneity test P was very small, and the I^2^ was larger (I^2^ threshold value: 50%).*

c *The sample size is small, and the CI is wide.*

d *Funnel plot was not symmetrical, or the number of included studies was small and all were positive results (sample size threshold value: 300)*.

### Efficacy and Safety of Moxibustion for KOA

#### Efficacy Evaluation

We performed a comprehensive analysis of the seven primary outcomes, as at least two SRs assessed these measures. The seven SRs (Song et al., 2016; Wang et al., 2017; Zhang et al., 2017; Li et al., 2019; Lu et al., 2019; Deng et al., 2020; Zhang et al.) reported that the total effectiveness rate is better in the treatment group than in the control group (*P* < 0.05). Compared with the control group, the treatment group of five SRs (Ma et al., 2017; Wang et al., 2017; Li et al., 2019; Deng et al., 2020; Zhang et al.) had a better reduction in pain score (*P* < 0.05), the treatment group of three SRs (Li et al., 2019; Lu et al., 2019; Zhang et al.) had a lower WOMAC pain score (*P* < 0.05) and the treatment group of two SRs (Ma et al., 2017; Zhang et al.) had a lower WOMAC scale score (*P* < 0.05). The Lysholm score of three SRs (Wang et al., 2017; Li et al., 2019; Lu et al., 2019) and the Lequesne index of two SRs (Li et al., 2019; Deng et al., 2020) in the treatment group were significantly different from that in the control group (*P* < 0.05). However, the SF-36 scale showed no significant difference between the treatment group and the control group (Li et al., 2016; Ma et al., 2017). More results are shown in Table 5.

#### Results of the Meta-Analysis

**Figure 3:**
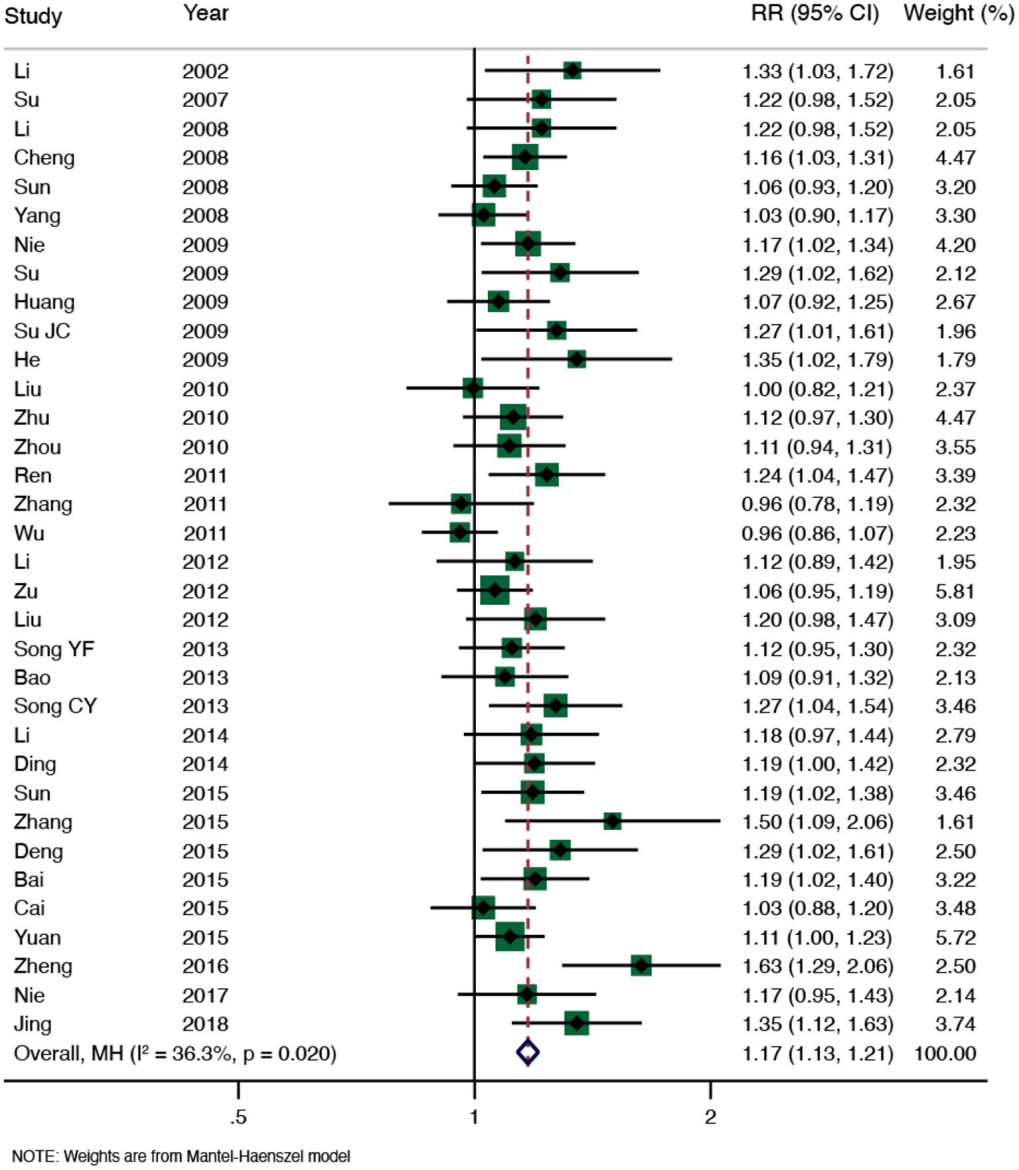
Meta-analysis of total effectiveness rate (moxibustion for KOA).

**Figure 4:**
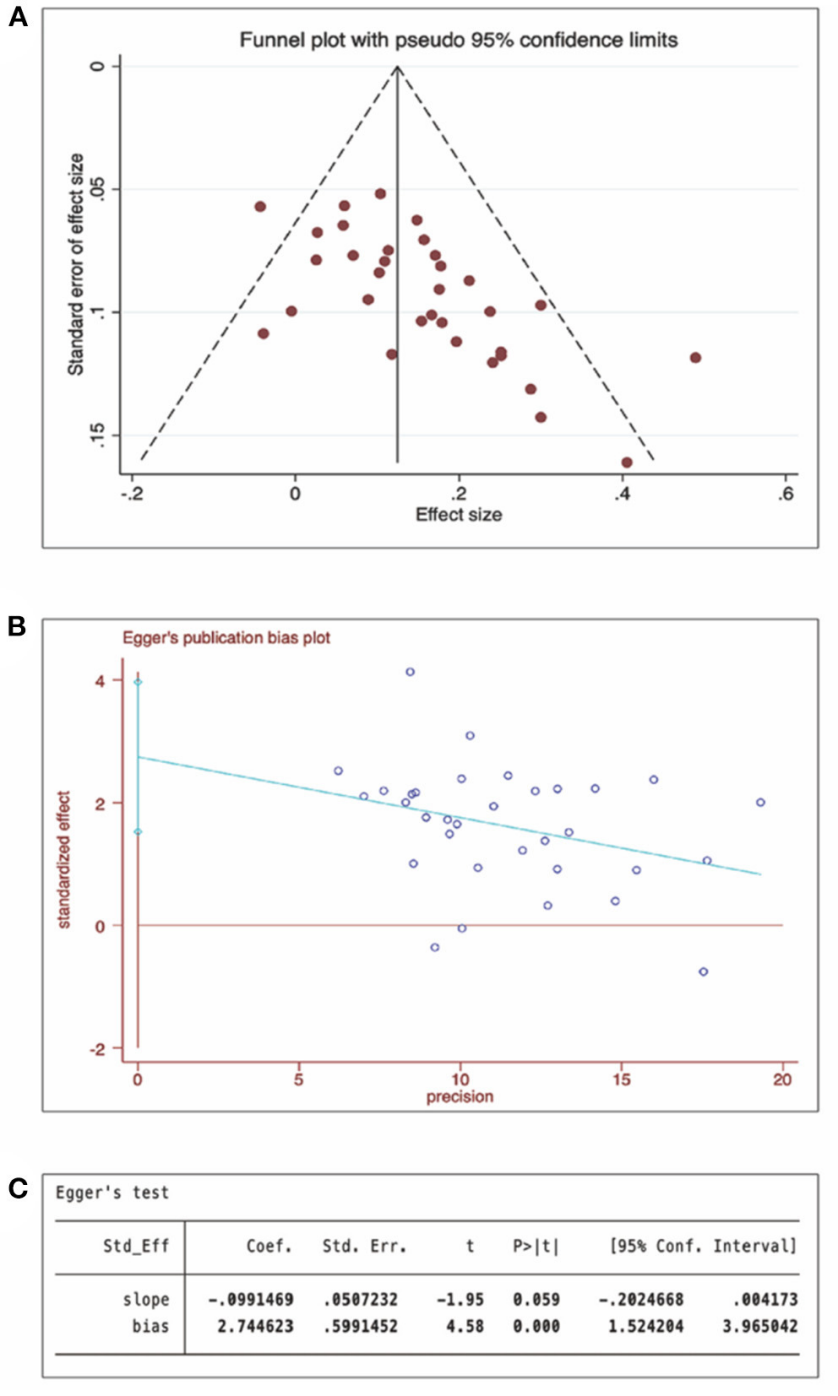
Publication bias of total effectiveness rate (moxibustion for KOA). **(A)** Funnel plot, **(B)** Egger's test, and **(C)** Egger's test *P-*value.

**Figure 5:**
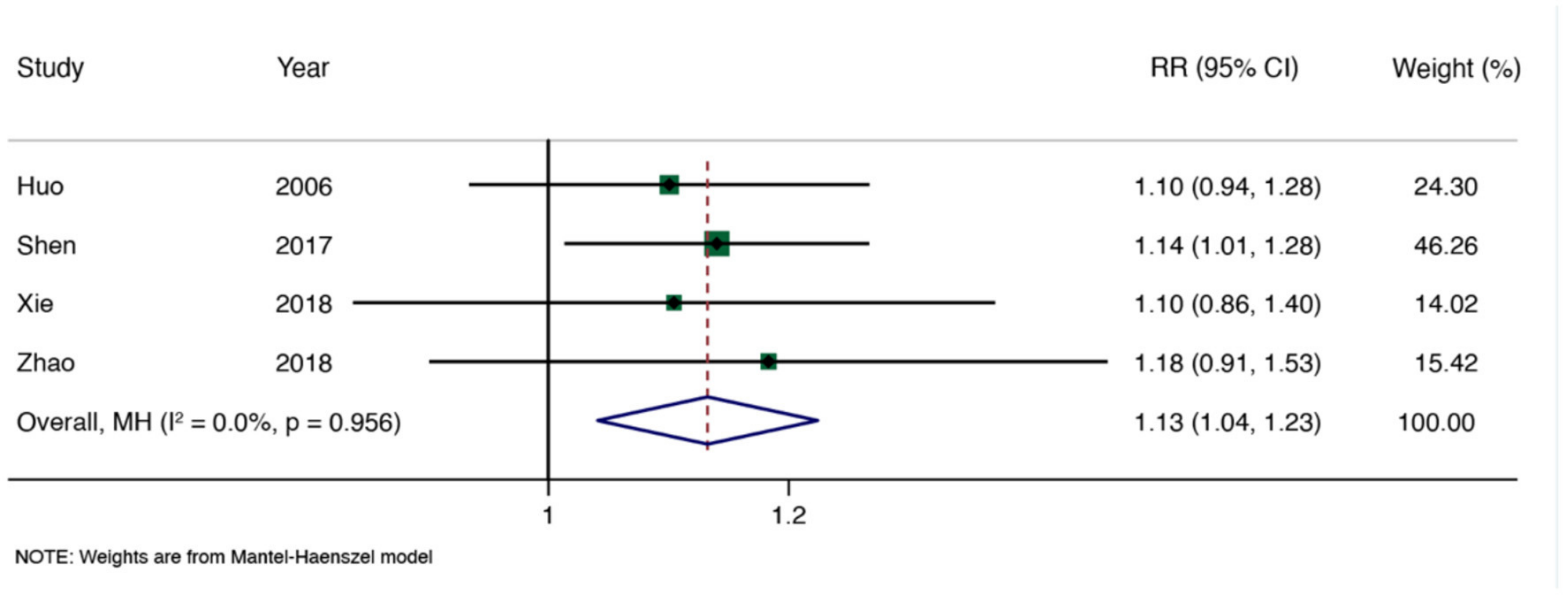
Meta-analysis of total effectiveness rate (moxibustion combined treatment for KOA).

We conducted a meta-analysis on the total effectiveness rate (Song et al., 2016; Wang et al., 2017; Zhang et al., 2017; Fan et al., 2018; Li et al., 2019; Lu et al., 2019; Deng et al., 2020; Zhang et al.). A total of 34 RCTs (2,828 participants) were included after duplicates were removed, meta-analysis results of moxibustion for KOA showed significant homogeneity among all studies (*I*^2^ = 36.3%, *P* = 0.020), the effect of moxibustion on the total effectiveness rate of KOA was better than that of the control group (RR = 1.17, 95% CI: 1.13–1.21, and *P* < 0.001; Figure 3). The funnel plot and Egger's test showed obvious publication bias (Figure 4), and the sensitivity analysis showed that the results are stable (Supplementary Material 1). The results of the moxibustion combined treatment for KOA showed no heterogeneity (*I*^2^ = 0.0%, *P* = 0.956), moxibustion combined treatment group could better improve the total effectiveness rate of KOA patients compared with the control group (RR = 1.13, 95% CI: 1.04–1.23, *P* < 0.001, 4 RCTs, and 360 participants; Figure 5).

#### Adverse Events

A total of eight SRs mentioned adverse events, among which four SRs (Li et al., 2016; Song et al., 2016; Zhang et al., 2017; Lu et al., 2019) reported common discomfort symptoms such as blisters, skin flushing, burn wounds, rash and itching, caused by moxibustion. One SR (Zhang et al., 2017) indicated that the symptoms of moxibustion were milder relative to the control group (*P* < 0.01). Another SR (Deng et al., 2020) conducted a meta-analysis of adverse events, and the results showed that the moxibustion group did not show significant differences when compared with the control group (*P* > 0.05).

## Discussion

### Summary of the Main Results

This overview comprehensively evaluated the available evidence from 10 different SRs on the efficacy and safety of moxibustion for KOA and evaluated the quality of methodology and evidence. In the PRISMA checklist, the quality of SRs was relatively good, and the manuscript structures were relatively complete. However, in the GRADE results, the evidence quality was poor, and all SRs evaluated by AMASTAR-2 had more than one critical deficiency, so all SRs were rated as critically low. AMSTAR-2 was updated in 2017, and the two SRs (Li et al., 2016; Song et al., 2016) before that may have biased the evaluation results because they did not conceal some items of AMSTAR-2. Six SRs were rated as having high RoB using the ROBIS tool. Finally, we performed an updated meta-analysis on the total effectiveness rate of the original study. Evidence shows that the moxibustion and moxibustion combined treatment of KOA has a higher total effectiveness rate than the control treatment, and the heterogeneity is low. Although moxibustion and moxibustion combined treatment showed consistent results, the small effect size indicated that the effect was not very significant, and more original studies of higher quality are needed to support this result in the future.

### Results-Based Discussion

While all SRs appeared to show the benefits of moxibustion, the results of the comprehensive overview were not ideal. We considered two main reasons: (a) The author's report on SR was incomplete, and the neglect of some items directly led to the degradation of the methodological quality. Key items such as early registration protocol, detailed exclusion list and reasonable explanation of bias risk are not stated in the SR. (b) To trace the root of the issue, the quality of the original research was the basis for determining the quality of the SR evidence. We found that each RCT used different criteria. For example, the inclusion criteria of the patients, evaluation of patients' condition, setting of the control group, selection of observation indicators, and other reasons may lead to bias. When RCTs of different criteria are included, the RoB in SR may increase, with high heterogeneity, and precise research results cannot be obtained, which also reduces the credibility of evidence for the moxibustion treatment of KOA to some extent. Therefore, we make the following important suggestions: (a) Regardless of whether it is SR or RCT, it is necessary to follow the relevant literature reporting guidelines [such as the PRISMA checklist (Zhang et al., 2020) and CONSORT statement (Cheng et al., 2013)]. (b) The adoption of internationally agreed diagnostic criteria and the evaluation criteria for effectiveness may reduce heterogeneity. (c) Moxibustion is a non-invasive treatment method, and sham moxibustion devices are often used to verify the specificity of moxibustion in clinical practice. Although sham moxibustion can reduce the skin irritation caused by its warm effect as much as possible (Zhao et al., 2006; Kim et al., 2011), it may still bring false-positive results. Therefore, researchers should pay more attention to the long-term efficacy of moxibustion in the treatment of KOA, increase the follow-up time and verify the specific therapeutic effect of moxibustion. Of the 10 SRS included in this study, a few reported available data during follow-up, and we encourage better study design to maximize scientific evidence of the effectiveness of moxibustion for the treatment of KOA.

### Selection of KOA Outcomes

As regards outcomes, pain, knee function, and total effectiveness rate are commonly used outcomes in the study of KOA. In evaluating the improvement of KOA pain, visual analog scale (VAS), and numerical rating scale are the two most used scales with excellent retest reliability. Among them, VAS has the highest reliability in measuring KOA pain. Evidence showed that the two scales demonstrated a good correlation (Alghadir et al., 2018). For better statistical analysis, we combined the two indexes into the pain score. WOMAC, Lysholm score, Lequesne index, and SF-36 scale are comprehensive assessment scales with different emphasis. The WOMAC scale is divided into pain, stiffness, and body function, which can effectively evaluate the course and treatment effect in patients with KOA and is widely used (Xie et al., 2008; Collins et al., 2011). The Lysholm score and Lequesne index are commonly used to evaluate the knee function of patients with KOA with high reliability (Nilsdotter and Bremander, 2011; Lecorney et al., 2018; Ahmed et al., 2019). As a general quality of life self-assessment scale, the SF-36 scale is widely used. In this study, two SRs (Li et al., 2016; Ma et al., 2017) reported this outcome, and the results showed that moxibustion had no significant difference in improving the quality of life of patients with KOA compared with the control group. However, whether the SF-36 scale can be used as an evaluation standard for moxibustion to improve the quality of life of patients with KOA remains to be further explored (Li et al., 2003; Lins and Carvalho, 2016). Inconsistent diagnostic criteria for SRs may lead to inconsistent assessment criteria for effectiveness, ultimately affecting the reliability of results.

### Mechanism of Moxibustion in the Treatment of KOA

The traditional Chinese medicine theory believes that the efficacy of moxibustion is based on two aspects: the role of meridians and moxa fire. KOA is one of the common indications for moxibustion (Huang et al., 2012a), and its analgesic mechanism may involve the thermal, radiation and pharmacological effects of moxibustion and its combustion products (Zhu et al., 2017). In addition, moxibustion treatment of KOA involves multiple inflammatory signaling pathways (Zhang et al., 2021). It is also associated with cytokines, matrix metalloproteinases, chondrocytes and other factors (He et al., 2017). Pain is the primary reason for patients with KOA to seek medical attention. Arthritis pain and functional limitation seriously affect the quality of life of patients. Therefore, most studies have regarded the improvement of moxibustion on inflammatory knee pain as the main outcome. Based on existing evidence, moxibustion has great prospects in relieving KOA pain and improving joint function, but its effectiveness still needs to be confirmed by more high-quality RCTs.

Moxibustion is a double-edged sword. Manifestations such as burns, itching, fatigue, blisters, and skin flushing resulting from the moxibustion process are not only the factors of therapeutic effect of moxibustion, but also the main contributing factors of adverse events. However, these adverse events can spontaneously subside, even adverse events such as burns and blisters can be avoided and most patients can accept moxibustion treatment (Ren et al., 2015). The main product of moxibustion is moxa smoke, functioning as antibacterial disinfection, increasing immunity, anti-aging, and regulating blood lipids. However, the concentrations of mono-aromatic hydrocarbons, formaldehyde, and polycyclic aromatic hydrocarbons produced by moxa smoke seriously exceed the standard, which also causes harm to the human body and environment (Mo et al., 2014; Deng et al., 2021). In the main biological pathway of toxic chemical components, high concentrations of moxa smoke are toxic to a certain extent to the heart, liver, and kidney. However, there are no clinical reports of related toxic and side effects; thus, the characteristics of the clinical application of traditional Chinese medicine should be considered comprehensively instead of discussing its toxicity in isolation (Xu, 2021). An RCT compared smokeless moxibustion with conventional moxibustion in the treatment of KOA and showed that moxa smoke did not affect the efficacy of moxibustion in the treatment of KOA but may be limited to patients with moderate KOA pain or functional limitations (Luo et al., 2019). Essentially, the effectiveness and safety of moxibustion for KOA should be fully considered. For patients with KOA of different severities, its effectiveness should be verified. Among the 10 SRs evaluated in this study, we did not obtain data on the patient's condition. From the existing evidence, we still support moxibustion as an effective and safe method to treat KOA.

### Strengths and Limitations

This overview comprehensively searched relevant literature and comprehensively evaluated the effectiveness and safety of moxibustion in the treatment of KOA by including different moxibustion treatments of KOA based on existing evidence. We reported the quality of reporting, methodological quality, and RoB for SRs using the AMSTAR-2, PRISMA, and ROBIS assessment tools and assessed the quality of evidence of clinical outcomes using GRADE. All assessments involved at least two independent reviewers, thus ensuring the reliability of the overview results as far as possible. Finally, we reconstructed the total effectiveness rate of individual RCTs included in all SRs and showed that acupuncture was effective in treating KOA with stable results.

The study designs of the original literature varied, and the evaluation results of SRs were highly heterogeneous, which may be the primary reason for the degradation of the outcomes. Secondly, the RoB assessment in this study was conducted for a single SR, and we were unable to retrieve all available data from the original study, and the results may not be comprehensive enough. Therefore, this may have hindered the overall evaluation of this study.

## Conclusion

This overview suggests that moxibustion seems to be effective in treating KOA. However, the reliability of the results is reduced by the high RoB of the original studies and the low methodological quality of SRs. Thus, we continue to support the value of moxibustion as a non-invasive treatment for KOA. Future studies should pay more attention to the quality of original studies and evidence quality of SR to provide more powerful and scientific evidence for the effectiveness and safety of moxibustion in the treatment of KOA.

## Data Availability Statement

The original contributions presented in the study are included in the article/Supplementary Material, further inquiries can be directed to the corresponding author.

## Author Contributions

SY and FZ: study conception and design. LL and DC: administrative support. ZL, JF, LZ, and ZH: collection and assembly of data. SY and ZL: data analysis and interpretation. SY and FZ: manuscript writing. All authors: final approval of manuscript. All authors contributed to the article and approved the submitted version.

## Conflict of Interest

The authors declare that the research was conducted in the absence of any commercial or financial relationships that could be construed as a potential conflict of interest.

## Publisher's Note

All claims expressed in this article are solely those of the authors and do not necessarily represent those of their affiliated organizations, or those of the publisher, the editors and the reviewers. Any product that may be evaluated in this article, or claim that may be made by its manufacturer, is not guaranteed or endorsed by the publisher.
