# Supplementary material for: An Overview of Systematic Reviews of Moxibustion for Knee Osteoarthritis
Source: Front Physiol. 2022 Feb 3;13:822953. doi: 10.3389/fphys.2022.822953 (PMC8850775; doi:10.3389/fphys.2022.822953)

Search strategy sample of PubMed

#1	osteoarthritis, knee [MeSH terms]
#2	Osteoarthritis [MeSH terms]
#3	knee osteoarthritis [text word]
#4	KOA [text word]
#5	OA [text word]
#6	Or/#1-5
#7	Moxibustion [MeSH terms]
#8	systematic review [text word]
#9	systematic evaluation [text word]
#10	meta-analysis [text word]
#11	Or/#8-10
#12	#6 and #7 and #11
(("osteoarthritis, knee"[MeSH Terms] OR "Osteoarthritis"[MeSH terms] OR "knee osteoarthritis"[All Fields] OR "KOA"[All Fields] OR "OA"[All Fields]) AND ("Moxibustion"[MeSH Terms]) AND ("systematic review"[All Fields] OR "systematic evaluation"[All Fields] OR "meta-analysis"[All Fields]))	

Search strategy sample of CNKI

SU=(’膝骨关节炎’+’膝关节骨性关节炎’+’骨关节炎’+’KOA’+’膝关节骨关节炎’+’膝关节炎’+’骨性关节炎’+’膝退行性变’+'老年性骨性关节炎'+'膝痹'+'骨痹') AND SU=(’艾灸’+’艾灸治疗’+’灸’+’艾柱灸’+’艾条灸’+’艾灸疗法’+’温和灸’+’热敏灸’+’雷火灸’+’隔物灸’) AND SU=(’系统综述’+’系统评价’+’系统’+’Meta 分析’+’荟萃分析’+’汇总分析’+’集成分析’+’二次分析’)

Excluded list

Citation	Reason for exclusion
Yu H, Wang Y, Guo Y, Wang H, Chen B, Zhao X. Quality assessment of randomized controlled trials reporting on knee osteoarthritis treated with warming needle moxibustion. J Tradit Chin Med. 2014;34(5):621-626.	Not systematic review
Ferreira RM, Duarte JA, Gonçalves RS. Non-pharmacological and non-surgical interventions to manage patients with knee osteoarthritis: An umbrella review. Acta Reumatol Port. 2018;43(3):182-200.	Not systematic review
Li J, Li YX, Luo LJ, et al. The effectiveness and safety of acupuncture for knee osteoarthritis: An overview of systematic reviews. Medicine (Baltimore). 2019;98(28):e16301.	Not systematic review
Qin S, Chi Z, Xiao Y, et al. Effectiveness and safety of massage for knee osteoarthritis: A protocol for systematic review and meta-analysis. Medicine (Baltimore). 2020;99(44):e22853.	Not systematic review
Wang X, Jiang Y, Xiong J, et al. Moxibustion for treating knee osteoarthritis: A protocol for systematic review and meta analysis. Medicine (Baltimore). 2020;99(19):e19974.	Not systematic review
Cheng S, Zhou J, Xu G, et al. Acupuncture and moxibustion for pain relief and quality of life improvement in patients with knee osteoarthritis: A protocol for systematic review and meta-analysis. Medicine (Baltimore). 2020;99(22):e20171.	Not systematic review
Yuan T, Xiong J, Wang X, et al. The Quality of Methodological and Reporting in Network Meta-Analysis of Acupuncture and Moxibustion: A Cross-Sectional Survey. Evid Based Complement Alternat Med. 2021;2021:2672173.	Not systematic review
Huang Q, Chen J, Jiang Y, et al. Efficacy and safety of thunder-fire moxibustion for patients with knee osteoarthritis: A protocol for systematic review and meta-analysis. Medicine (Baltimore). 2021;100(14):e25384.	Not systematic review

Shen C, Li N, Chen B, et al. Thermotherapy for knee osteoarthritis: A protocol for systematic review. Medicine (Baltimore). 2021;100(19 Not systematic review Not systematic review Not systematic review): e25873.	Not systematic review
Jian-Hui, Sun et al. Zhongguo Zhong yao za zhi = Zhongguo zhongyao zazhi = China journal of Chinese materia medica vol. 45,17 (2020): 4065-4070.	Not systematic review
Liu W, Fan Y, Wu Y, et al. Efficacy of Acupuncture-Related Therapy in the Treatment of Knee Osteoarthritis: A Network Meta-Analysis of Randomized Controlled Trials. J Pain Res. 2021;14:2209-2228.	The main intervention is not moxibustion
Qu B, Wu X, Liu H, et al. Meta-analysis and systematic review of acupotomy combined with puncture and moxibustion in the treatment of knee osteoarthritis. Ann Palliat Med. 2021;10(6):6637-6649.	The main intervention is not moxibustion
Park YC, Goo BH, Park KJ, Kim JY, Baek YH. Traditional Korean Medicine as Collaborating Treatments with Conventional Treatments for Knee Osteoarthritis: A Protocol for a Systematic Review and Meta-Analysis. J Pain Res. 2021;14:1345-1351.	The main intervention is not moxibustion
Zhang Q, Yue J, Golianu B, Sun Z, Lu Y. Updated systematic review and meta-analysis of acupuncture for chronic knee pain. Acupunct Med. 2017;35(6):392-403.	The main intervention is not moxibustion
Wang Y, Xie X, Zhu X, et al. Fire-Needle Moxibustion for the Treatment of Knee Osteoarthritis: A Meta-Analysis. Evid Based Complement Alternat Med. 2016;2016:1392627.	The main intervention is not moxibustion
Ferreira RM, Torres RT, Duarte JA, Gonçalves RS. Non-Pharmacological and Non-Surgical Interventions for Knee Osteoarthritis: A Systematic Review and Meta-Analysis. Acta Reumatol Port. 2019;44(3):173-217.	The main intervention is not moxibustion
Sun N, Tu JF, Lin LL, et al. Correlation between acupuncture dose and effectiveness in the treatment of knee osteoarthritis: a systematic review. Acupunct Med. 2019;37(5):261-267.	The main intervention is not moxibustion
Liu Y, Xu J, Lin M. Systematic evaluation and meta-analysis of moxibustion therapy for knee osteoarthritis. Journal of Clinical and Experimental Medicine.2017;(08) :783-787.	Repeated publications

Wang Y. Systematic evaluation of clinical efficacy of moxibustion therapy in patients with knee osteoarthritis. Health for All.2019;(24):12.	Unable to extract data
Choi TY, Choi J, Kim KH, Lee MS. Moxibustion for the treatment of osteoarthritis: a systematic review and meta-analysis. Rheumatol Int. 2012;32(10):2969-2978.	The main disease is not KOA
Choi TY, Lee MS, Kim JI, Zaslawski C. Moxibustion for the treatment of osteoarthritis: An updated systematic review and meta-analysis. Maturitas. 2017;100:33-48.	The main disease is not KOA
Choi TY, Kim TH, Kang JW, Lee MS, Ernst E. Moxibustion for rheumatic conditions: a systematic review and meta-analysis. Clin Rheumatol. 2011;30(7):937-945.	The main disease is not KOA
Yuan T, Xiong J, Wang X, et al. The Effectiveness and Safety of Moxibustion for Treating Knee Osteoarthritis: A PRISMA Compliant Systematic Review and Meta-Analysis of Randomized Controlled Trials. Pain Res Manag. 2019;2019:2653792.	Control group with moxibustion
Yu J, Xiong J. Systematic evaluation and meta-analysis of clinical efficacy of thermo-sensitive moxibustion in treatment of knee osteoarthritis. Journal of Guangzhou University of Traditional Chinese Medicine. 2015;(01):60-66.	Control group with moxibustion
Tao S, Zheng J, Liang F, et al. Systematic evaluation and meta-analysis of thermosensitive moxibustion in the treatment of knee osteoarthritis. Modern Preventive Medicine. 2017;(21):4027-4032.	Control group with moxibustion

Sensitivity analysis:

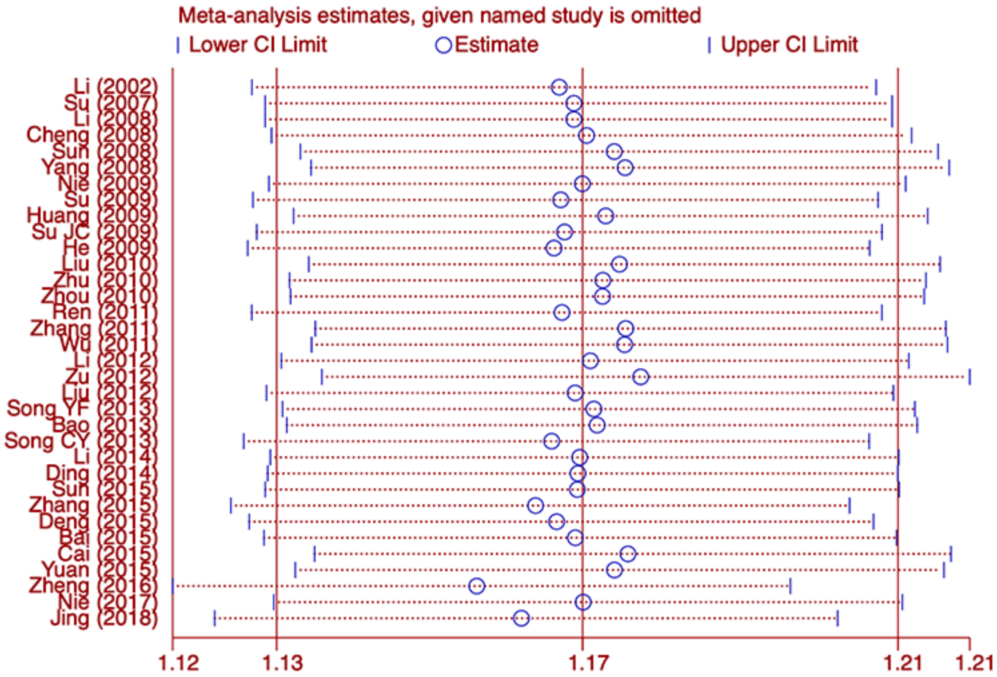

Supplement: Supplementary file 1 [file Data_Sheet_1.PDF]
