# Supplementary material for: An Overview of Systematic Reviews of Moxibustion for Knee Osteoarthritis
Source: Front Physiol. 2022 Feb 3;13:822953. doi: 10.3389/fphys.2022.822953 (PMC8850775; doi:10.3389/fphys.2022.822953)
Supplement: Supplementary file 2 [file Data_Sheet_2.PDF]

Citations list of included RCTs (references in bold red were used for meta-analysis).

1. Chen R, Chen M, Su T, Zhou M, Sun J, Xiong J, et al. Heat-sensitive moxibustion in patients with osteoarthritis of the knee: a three-armed multicentre randomised active control trial. *Acupunct Med* (2015) 33(4):262-9. Epub 2015/05/23. doi: 10.1136/acupmed-2014-010740. PubMed PMID: 25998755.
2. Dai M, Fang X-y, Chen H-y, Wang Y-h, Wu Y-w. Clinical study on mild moxibustion for knee osteoarthritis. *Journal of Acupuncture and Tuina Science* (2019) 17(1):62-6. doi: 10.1007/s11726-019-1084-0.
3. Huang Z, Song S-l. Observation on clinical effects of herbal cake-partitioned moxibustion for knee osteoarthritis. *Journal of Acupuncture and Tuina Science* (2015) 13(4):242-5. doi: 10.1007/s11726-015-0860-8.
4. Kim TH, Kim KH, Kang JW, Lee M, Kang KW, Kim JE, et al. Moxibustion treatment for knee osteoarthritis: a multi-centre, non-blinded, randomised controlled trial on the effectiveness and safety of the moxibustion treatment versus usual care in knee osteoarthritis patients. *PLoS One* (2014) 9(7):e101973. Epub 2014/07/26. doi: 10.1371/journal.pone.0101973. PubMed PMID: 25061882; PubMed Central PMCID: PMCPMC4111481.
5. Ren X, Yao C, Wu F, Li Z, Xing J, Zhang H. Effectiveness of moxibustion treatment in quality of life in patients with knee osteoarthritis: a randomized, double-blinded, placebo-controlled trial. *Evid Based Complement Alternat Med* (2015) 2015:569523. Epub 2015/02/18. doi: 10.1155/2015/569523. PubMed PMID: 25688277; PubMed Central PMCID: PMCPMC4320789.
6. **Sun K, Yang J, Shen DK. Clinical observation on treatment of primary knee osteoarthritis of liver and kidney deficiency type with Aconite cake-separated moxibustion. *Zhongguo Zhen Jiu* (2008) 28(2):87-90. Epub 2008/04/15. PubMed PMID: 18405148.**
7. Zhao L, Cheng K, Wang L, Wu F, Deng H, Tan M, et al. Effectiveness of moxibustion treatment as adjunctive therapy in osteoarthritis of the knee: a randomized, double-blinded, placebo-controlled clinical trial. *Arthritis Res Ther* (2014) 16(3):R133. Epub 2014/06/26. doi: 10.1186/ar4590. PubMed PMID: 24962039; PubMed Central PMCID: PMCPMC4095686.
8. **Ding Hongmei. Effect of septum moxibustion, TDP irradiation and nursing intervention on the curative effect of knee osteoarthritis. *Clinical Journal of Traditional Chinese Medicine* (2014) 26(6):617-8.**
9. **Ren Xiu-mei, CAO Jin-Jin, SHEN Xue-yong, WANG Li-Zhen, Zhao Ling, WU Fan, et al. Moxibustion for knee osteoarthritis: a randomized controlled study. *Chinese acupuncture* (2011) 31(12):1057-61.**
10. **He Yong, XU Yangping, HUO Xiongtao. Clinical study of moxibustion on knee osteoarthritis. *Chinese Journal of Orthopedics and Traumatology* (2009) 17(3):38-9.**
11. **Liu Dechun, Chu Zhigao. Treatment of 42 cases of knee osteoarthritis with sappanwood decoction and moxibustion. *Chinese Medicine Guide* (2012) 18(7):104-5. doi: 10.3969/j.issn.1672-951X.2012.07.052.**
12. **Liu Hui. Clinical study of thunder fire moxibustion in the treatment of knee osteoarthritis [Master's]: Beijing University of Chinese Medicine (2010).**
13. Liu Hongyan, Zhu Huan. Clinical observation of thunder fire moxibustion combined with

Sanfu paste in treatment of knee osteoarthritis with cold coagulation of Yang deficiency. *Clinical study of Chinese Medicine* (2017) 9(18):74-6.

14. Wu Feng, XIONG Peng. Clinical study on moxibustion heat sensitization acupoints in the treatment of knee osteoarthritis (swelling type). *Clinical Journal of Acupuncture and Moxibustion* (2011) 27(11):1-4. doi: 10.3969/j.issn.1005-0779.2011.11.001.

15. Zhou Zhong-liang, Sun Kui, Cheng Hong-liang, Liu De-chun, Yang Jun. Clinical observation on the treatment of knee osteoarthritis with blood stasis by moxibustion. *Shanghai Journal of Acupuncture and Moxibustion* (2010) 29(1):45-7.

16. Zhou Yanli, Li Jing, Hou Wenguang, Bao Chunling, Zhang Qian, Wang Shuoshuo, et al. Clinical observation of moxibustion in treatment of knee osteoarthritis. *Shanghai Journal of Acupuncture and Moxibustion* (2014) (12):1086-8. doi: 10.13460/j.issn.1005-0957.2014.12.1086.

17. Sun Li-ming, SUN Shi-jie, SUN Li-Hong, LIANG Yu-lei, ZHANG Xuan-ping, LI Jie, et al. Study on the curative effect of ear acupuncture and moxibustion on knee osteoarthritis with cold coagulation of Yang Deficiency. *Hebei Journal of Traditional Chinese Medicine* (2015) (1):44-6,7.

18. Song Yofen. Observation and nursing of Chinese herbal fumigation combined with moxibustion in the treatment of knee osteoarthritis. *Jilin medical* (2013) 34(32):6858-9.

19. Song Yangchun, Liu Dechun, Zhu Junchen. Clinical study on the treatment of knee osteoarthritis with blood stasis by moxibustion every thirty-seven cakes. *Clinical Journal of Acupuncture and Moxibustion* (2013) (9):40-2. doi: 10.3969/j.issn.1005-0779.2013.09.015.

20. Chang Wen-yan, Liu Jie, Liang Jian-hua. Effect of thunder fire moxibustion combined with ultrashort wave on knee osteoarthritis. *Liaoning Journal of Traditional Chinese Medicine* (2013) 40(09):1857-8.

21. Zhang Qianjin, Cao Lihu, Li Zhuodong, Wang Sicheng, Ma Yuhai, SU Jiacan, et al. Clinical effect and safety of moxibustion and celecoxib in the treatment of knee osteoarthritis. *Chinese Journal of Orthopedics and Traumatology* (2011) 19(1):13-5.

22. Zhang Huajun, Xu Haidong, LIU Tingting, Li Peifa. Clinical study of electroacupuncture combined with thunder and fire moxibustion in treatment of degenerative knee arthritis with cold and dampness obstruction. *Chinese acupuncture* (2016) 36(12):1266-70. doi: 10.13703/j.0255-2930.2016.12.011.

23. Zhang Hanyi, Xu Daoming, Xue Liang, Li Yongjun, PANG Gensheng, Wang Hesheng, et al. Clinical study of thunder fire moxibustion combined with celecoxib in the treatment of knee osteoarthritis. *Journal of Nanjing University of Traditional Chinese Medicine* (2017) 33(6):574-8. doi: 10.14148/j.issn.1672-0482.2017.0574.

24. Zhang Yang-chun, Li Xiu-bin. Clinical observation on the treatment of 30 cases of knee osteoarthritis with "Shuang-gu-yi" septum and mild moxibustion. *Zhejiang Journal of Traditional Chinese Medicine* (2015) 50(05):382.

25. Jing Hongbo, Qin Wanyu, Deng Xiaohong. Clinical observation of mild moxibustion combined with intraarticular injection of sodium hyaluronate in treatment of 60 cases of knee osteoarthritis. *Clinical Journal of Traditional Chinese Medicine* (2018) 30(02):335-7.

26. Zhu Ying, Chen Iran, Miao Furui, Ji Le. Therapeutic effect of moxibustion combined with electroacupuncture on knee osteoarthritis of cold and wet type. *Acupuncture*

research (2010) 35(4):293-7.

27. Li Ning, Wu Bin, Zhang Yongling. Effect of moxibustion combined with exercise therapy on knee osteoarthritis. *Chinese acupuncture* (2002) 22(11):729-31.

28. Li Jianwu, Xiang Shiyu, Ma Zhiyi, Feng Yanbin, Tong Huiyun, Geng Huiping, et al. Clinical observation on the treatment of knee osteoarthritis with septum and moxibustion. *Chinese acupuncture* (2008) 28(1):17-9.

29. Li Si-bin, YUAN Hui, LI Fei, LI Zheng-xiang. Clinical observation on the treatment of primary osteoarthritis of knee with blood stasis by moxibustion and massage every thirty-seven cakes. *Journal of Zhejiang Traditional Chinese Medicine University* (2014) 38(05):631-4.

30. Li Yanling. Clinical effect of moxibustion on knee osteoarthritis. *The Chinese and foreign medical* (2012) 31(2):130. doi: 10.3969/j.issn.1674-0742.2012.02.100.

31. Li Jingkun, GAO Yajing, Shi Zhimin, Xu Zhiguo, HAO Xiuyuan. Effect of thunder fire moxibustion combined with ozone therapy on knee osteoarthritis and its effect on serum inflammatory factors. *Journal of Guangzhou University of Traditional Chinese Medicine* (2020) 37(10):1950-5. doi: 10.13359/j.cnki.gzxbtcm.2020.10.021.

32. Yang Yonghui, Sun Kui, SU Guohong, ZHOU Zhongliang. Clinical study on the treatment of primary knee osteoarthritis with qi stagnation and blood stasis by thirty-seven cakes moxibustion. *Clinical Journal of Traditional Chinese Medicine* (2008) 20(1):53-5.

33. Bai Wenjie, Zou Zhuocheng. Observation on the curative effect of moxibustion warm passage therapy on knee osteoarthritis. *Shi Zhen Chinese medicine* (2015) 26(2):397-400. doi: 10.3969/j.issn.1008-0805.2015.02.057.

34. Shen Bo. Clinical effect of thunder fire moxibustion on 80 cases of knee osteoarthritis. *Health and Nutrition in China* (2017) 27(10):380. doi: 10.3969/j.issn.1004-7484.2017.10.588.

35. Teng Juzan, Su Bo, Wang Dawei. Arthroscopic debridement combined with thunder fire moxibustion to treat 30 cases of knee osteoarthritis with cold coagulation of Yang deficiency. *The doctor of traditional Chinese medicine in sichuan* (2013) 31(02):109-11.

36. Zu Na, ZHAO Cuifang, Guan Song, JI Zhenghan, HAO Gui-xiang, ZHU Yue-lan. Clinical study on the treatment of knee osteoarthropathy with thunder fire moxibustion. *Journal of Beijing University of Chinese Medicine (Clinical Edition of TCM)* (2012) 19(2):43-4. doi: 10.3969/j.issn.1672-2205.2012.02.014.

37. Cheng Hong-liang, Han Wei, Hu Pei-jia, Yang Jun. Clinical study of treating primary osteoarthritis of knee with septum moxibustion based on differentiation of symptoms. *Clinical Journal of Traditional Chinese Medicine* (2008) 20(2):114-6.

38. Nie Bin, Zhang Li, Xu Kai, Zhong Huiping. Clinical observation of Zhao's thunder moxibustion in treatment of knee osteoarthritis. *Jilin Traditional Chinese Medicine* (2009) 29(4):313-4. doi: 10.3969/j.issn.1003-5699.2009.04.023.

39. Nie Bin, Tan Yuan, Sun Zhengping, Yan Dongdong, Wang Yingshan, Xu Xuemeng, et al. Clinical observation of fuyang fire moxibustion in treatment of knee osteoarthritis with cold coagulation of Yang deficiency. *Rheumatism and arthritis* (2017) 6(3):22-5. doi: 10.3969/j.issn.2095-4174.2017.03.005.

40. Su Jican, CAO Lihu, Li Zhuodong, Wang Sicheng, ZHANG Qianjin, Ma Yuhai, et al. A

case - controlled trial of moxibustion in the treatment of knee osteoarthritis. *China bone injury* (2009) 22(12):914-6. doi: 10.3969/j.issn.1003-0034.2009.12.016.

41. Su Jiachan, Cao Lihu, Chen Weihua, Wang Sicheng, Ma Yuhai, ZHANG Qianjin. Clinical observation of moxibustion combined with manipulation in the treatment of knee osteoarthritis. *Journal of Tongji University (Medical Science)* (2009) 30(5):79-82.

42. Su Xia. Clinical observation of septum mild moxibustion in treatment of knee osteoarthritis with liver and kidney insufficiency and blood stasis [Ms]: Hubei University of Traditional Chinese Medicine (2007).

43. CAI Jing-zhou, DENG Jia-qin, LI Xiang-li, XUE Dan. Clinical observation of knee joint salt moxibustion in the treatment of knee joint osteoarthritis. *Journal of Liaoning University of Traditional Chinese Medicine* (2015) 17(7):157-60. doi: 10.13194/j.issn.1673-842x.2015.07.053.

44. Yuan Qing-dong, GUO Xin, HAN Ya-chen, ZHANG Jing-qian, FENG Xiao-dong. Effect of thunder fire - heat sensitive moxibustion on knee osteoarthritis. *Shanghai Journal of Acupuncture and Moxibustion* (2015) (7):665-8. doi: 10.13460/j.issn.1005-0957.2015.07.0665.

45. Qin Fei. Clinical observation on the treatment of knee osteoarthritis by thunder fire moxibustion combined with ozone cavity injection. *The light of traditional Chinese medicine* (2020) 35(5):728-32. doi: 10.3969/j.issn.1003-8914.2020.05.039.

46. Xie Mei-Fang, SHEN Si-yu, ZHOU Jin-bin, GAO Xue-qin. Clinical observation of thunder fire moxibustion and external application of Cold bi prescription combined with conventional Western medicine in treating 25 cases of wind-dampness-cold bi disease. *Rheumatism and arthritis* (2018) 7(12):12-5. doi: 10.3969/j.issn.2095-4174.2018.12.003.

47. Zhao Yonggang, Xu Yapeng. Clinical observation of aconitum decoction plus thunder fire moxibustion in treatment of cold and wet knee arthritis. *Modern Health Preservation (second half edition)* (2018) (8):116-7. doi: 10.3969/j.issn.1671-0223(x).2018.08.083.

48. Deng Jingming, Chen Ying, Wang Shengxu. Treatment of 35 cases of knee osteoarthritis by salt-separated moxibustion. *Clinical Journal of Acupuncture and Moxibustion* (2015) 31(03):14-7.

49. Deng Jingming, Chen Ying, Wang Shengxu. Comparative study on curative effect of warm moxibustion and salt-separated moxibustion on knee osteoarthritis. *Shanghai Journal of Acupuncture and Moxibustion* (2015) (3):243-5. doi: 10.13460/j.issn.1005-0957.2015.03.0243.

50. Zheng Wei, Gong Huahui. Clinical analysis of thunder fire moxibustion relieving pain of mild and moderate knee osteoarthritis. *World Clinical Medicine* (2016) 10(23):176-7.

51. Guo Yong-lin, Jiang Sheng-ping, FU Xiao-dam, Liu Qiong. Therapeutic effect of moxibustion combined with Chinese herbal fumigation on knee osteoarthritis. *Journal of Practical Chinese Medicine* (2013) 29(12):995-6. doi: 10.3969/j.issn.1004-2814.2013.12.011.

52. Zhao Haiyin, KONG Jingjing, LU Wang, Zhang Yan, Zhang Jun, Xu Jia, et al. Clinical effect of acupuncture on knee osteoarthritis. Journal of Shanghai University of Traditional Chinese Medicine (2013) 27(02):45-7. doi: 10.16306/ J.1008-861x.2013.02.015.

53. Huo Yongfang, Zhang Zhen, Ling Jun. Clinical observation of 60 cases of knee osteoarthritis treated by acupoint injection with thunder and fire moxibustion. *Chinese*

***Medicine Guide* (2006) (01):51-2.**

54. Ma Minglu. Clinical study of acupoint application of Thunder fire moxibustion combined with Dispelling cold and removing Stasis zhitong ointment in the treatment of knee osteoarthritis. *Integrated Traditional Chinese and Western Medicine Nursing (Chinese and English)* (2020) 6(8):106-8. doi: 10.11997/nitcwm.202008024.

55. Bao Xuemei, Sun Kui. Mechanism analysis of moxibustion with aconite cake in treating primary knee osteoarthritis with liver and kidney insufficiency. *Clinical Journal of Traditional Chinese Medicine* (2013) 25(09):797-8.

56. Huang Liqiang, JI Rong. Clinical study on the effect of thunder fire moxibustion on VAS and WOMAC score in patients with knee osteoarthritis. *Jiangsu Traditional Chinese Medicine* (2017) 49(8):57-8.

57. Huang Shu-hui, FENG Bi-jun, YU Peng, FAN Li, XU Zhen-hua. Clinical observation on the treatment of 35 cases of knee osteoarthritis by heat sensitive moxibustion. *New Traditional Chinese Medicine* (2009) 41(5):86-7.
